# The TNFSF Members APRIL and BAFF and Their Receptors TACI, BCMA, and BAFFR in Oncology, With a Special Focus in Breast Cancer

**DOI:** 10.3389/fonc.2020.00827

**Published:** 2020-06-16

**Authors:** Marilena Kampa, George Notas, Efstathios N. Stathopoulos, Andreas Tsapis, Elias Castanas

**Affiliations:** ^1^Laboratory of Experimental Endocrinology, School of Medicine, University of Crete, Heraklon, Greece; ^2^Laboratory of Pathology, School of Medicine, University of Crete, Heraklion, Greece

**Keywords:** APRIL (TNFSF13), BAFF (TNFSF13B), BCMA (TNFRSF17), TACI (TNFRSF13B), BAFFR (TNFRSF13C), breast cancer

## Abstract

Tumor necrosis factor (TNF) superfamily consists of 19 ligands and 29 receptors and is related to multiple cellular events from proliferation and differentiation to apoptosis and tumor reduction. In this review, we overview the whole system, and we focus on A proliferation-inducing ligand (APRIL, TNFSF13) and B cell-activating factor (BAFF, TNFSF13B) and their receptors transmembrane activator and Ca^2+^ modulator (CAML) interactor (TACI, TNFRSF13B), B cell maturation antigen (BCMA, TNFRSF17), and BAFF receptor (BAFFR, TNFRSF13C). We explore their role in cancer and novel biological therapies introduced for multiple myeloma and further focus on breast cancer, in which the modulation of this system seems to be of potential interest, as a novel therapeutic target. Finally, we discuss some precautions which should be taken into consideration, while targeting the APRIL–BAFF system.

## Introduction

The cytokine tumor necrosis factor (TNF) has been reported in 1968 as a cytotoxic factor, inducing tumor necrosis (1–3), while its sequence was described in 1984 (4) and its gene in humans was cloned in 1985 (5). The protein is produced as a 233-amino acid-long type II transmembrane protein (6–8), which is liberated in the extracellular space as a soluble homotrimer via proteolytic cleavage by the metalloprotease TNF-α converting enzyme (TACE/ADAM17) (9). TNF-α (TNFSF1B) and TNF-β (TNFSF1A) bind to two specific receptors (TNFR1 and TNFR2), which have been biochemically characterized in the late 80s (10–14) and were cloned in 1990 (15–17). In 1993, Banner et al. (18) reported the first ligand–receptor complex (PDB code 1TNR). In this structure, it is possible to identify a complex of a trimeric ligand with three receptor molecules, in which the cysteine-rich domains 2 and 3 of each receptor participate in ligand binding. In the same year, a characteristic structure in TNFR1 intracytoplasmic sequence, the death domain, was reported (19), which later was found to be responsible for the downstream signal transduction and the mediation of its cytotoxic effects (20). In the 90s, based on the structure of the TNF and the TNFR1/2, a number of other cytokines and receptor molecules, bearing functional moieties of the lead structures, were discovered. The TNF superfamily (TNFSF) includes now 19 molecules, while the TNF receptor superfamily (TNFRSF) comprises 29 distinct protein-members (21, 22). Apart from one-to-one selectivity between specific ligands and receptors, cross-reactivity has also been reported, suggesting a differential signaling and a plurality of putative cellular and molecular actions [see Figure 1 of Plitz et al. (20)].

TNF is considered a major pro-inflammatory and proapoptotic mediator; however, TNF expresses a functional duality, being involved in both tissue regeneration and destruction, indicative of an extreme diversity in signaling and phenotypic actions [reviewed in Wajant et al. (23)]. Classically, TNFR1 signaling proceeds: (1) through the association of TNFR1 with TRADD–TRAF–RIP initiating pathways leading to the nuclear factor (NF)-κB (canonical action) or c-Jun N-terminal kinase (JNK) or mitogen-activated protein kinase (MAPK)-p38 cascade activation; (2) through Fas-associated death domain (FADD) and pro-caspases-8 and -10.

**Figure 1:**
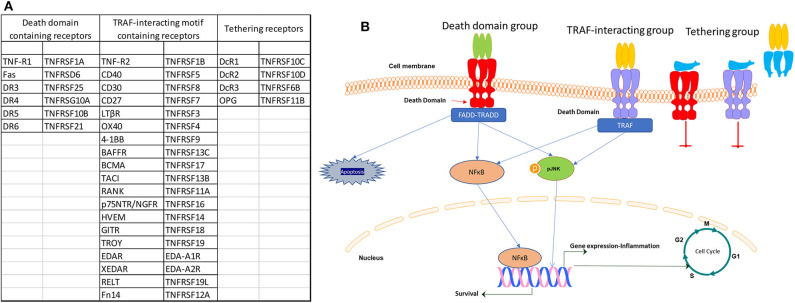
List **(A)** and signaling **(B)** of the three categories of TNF receptor superfamily (TNFRSF) members.

Apart from the death domain (DD)-containing TNFSF receptors, there is a group with receptors that contain TRAF-interacting motifs (TIMs) in their cytoplasmic part, which also includes B cell maturation antigen (BCMA, TNFRSF17), B cell-activating factor receptor (BAFFR, TNFRSF13C), and TNFR homolog transmembrane activator and Ca^2+^ modulator (CAML) interactor (TACI, TNFRSF13B) and another group with receptors which does not contain functional intracellular motifs and lack a direct ability for intracellular signaling. However, the later group competes with the other two receptor groups, either by tethering for ligand binding and functions by impeding signal transduction by other TNFRSF members (24). TNFSF receptors in each of the three categories and their schematic mode of action are presented in Figure 1. This plurality of structure–function has, therefore, a significant role in cell signaling and TNFSF actions, including cell survival and differentiation, cellular communication and cellular responses to inflammation and the identified multitude of actions in different tissues, from the regulation of immune cell activation and death to tissue homeostasis and cancer cell modulation.

In the present review, we will concentrate on the role of a proliferation-inducing ligand (APRIL, TNFSF13), BAFF (TNFSF13B), and their receptors in cancer, the novel biological therapies targeting this system, their role in breast cancer, and their potential use as therapeutic targets.

## APRIL, BAFF, and Their Receptor System: Identification, Signaling, and Cellular Actions

A proliferation-inducing ligand (APRIL or CD256, TNFSF13) (25–27) and B lymphocyte stimulator (BLyS), also known as BAFF (or TALL-1, CD257, TNFSF13B) (26, 28, 29), are relatively new additions to the TNFSF family. Both ligands bind to two TNFRSF members, TACI (TNFRSF13B) and BCMA (TNFRSF17) (30, 31), reviewed in Dillon et al. (32). However, reported affinities of ligands for each receptor differ significantly, with the affinity of BAFF for BCMA being 1,000 times lower than that of APRIL and APRIL affinity for TACI being one-third that of BAFF (33). BAFF also binds specifically to another TNFRSF member, BAFFR (TNFRSF13C). Apart from B cells, BAFF is expressed by monocytes, macrophages, dendritic cells, T cells (34–37), and neutrophils (38). As an addition to the complexity of the system, quite recently it was reported that membrane TACI and BCMA can be cleaved from the membrane, leading to soluble forms, which may act as decoy receptors, able to capture soluble BAFF and APRIL, and to decreased TACI and BCMA levels at the membrane (39, 40).

APRIL and BAFF play a differential role during B cell maturation and differentiation (41–43). They both promote B cell survival, maturation (28, 44), and differentiation through BCMA, which is increased during plasma cell differentiation, while BAFFR is decreased and is absent in plasma cells (45). BAFFR is important for B cell maturation (46, 47) as shown in knockout animals for BAFFR (48) and individuals with defective BAFFR, who do not have mature B cells (49, 50). Additionally, BAFF and APRIL, acting via TACI, have a regulatory effect on antibody production. *Taci*^−/−^ mice have reduced antibody titers, increased numbers of peripheral B cells, and hyperplasia of lymphoid organs (51). Indeed, TACI inhibits B cell proliferation, promotes their differentiation into plasma cells, and inhibits plasma cell apoptosis (52). Additionally, it plays a role on somatic hypermutation and immunoglobulin affinity maturation, as well as in T cell-dependent and T cell-independent antibody class switch recombination (53, 54).

**Figure 2:**
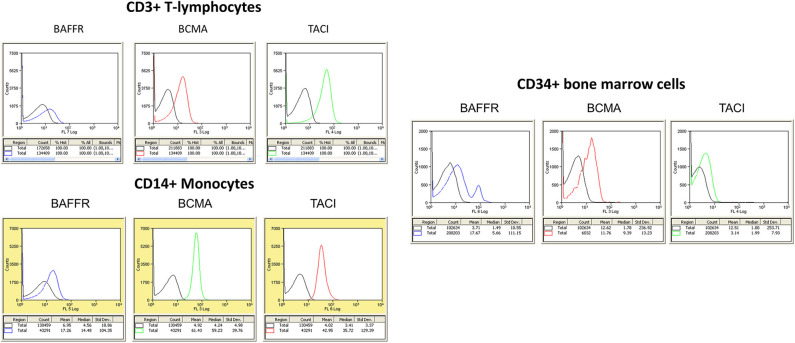
**(Left)** Expression of B cell maturation antigen (BCMA), TNFR homolog transmembrane activator and Ca^2+^ modulator (CAML) interactor (TACI), and B cell-activating factor receptor (BAFFR) in circulating human mononuclear cells. The different cell populations were isolated from peripheral blood mononuclear cells (PBMCs) using Ficoll-Hypaque gradient centrifugation. Whole mononuclear cell population was stained with specific antibodies, and populations were identified in flow cytometry with specific gating (CD3 for T lymphocytes and CD14 for monocytes). At least 5,000 events were collected and analyzed for each cell population. Results of flow cytometry in one human donor sample, repeated five times, with similar results. **(Right)** Expression of BCMA, TACI, and BAFFR in CD34+ hemopoietic blood cells. In both panels, negative isotype control is denoted by the black curve.

In addition to the expression of all three receptors in circulating B lymphocytes, as described above, BCMA and TACI are highly expressed on T lymphocytes (CD3+) and circulating monocytes (CD14+). Moreover, BCMA expression is the highest on CD34+ bone marrow hemopoietic progenitor cells (Figure 2). These data suggest an additional role of the BAFF–APRIL system in different normal circulating cell populations, beyond B lymphocytes. These data are in partial contradiction with other, previously published, observations. Indeed, in one of the scarce papers of APRIL–BAFF receptors in freshly prepared human monocytes, the authors report a negative membrane staining for either TACI or BCMA, while intracellular TACI translocated to the membrane, after BAFF or IL-10 incubation (55). A notable difference between the two studies is that in data presented here, no further purification of monocytes was performed, buffy-coat cells being directly stained, in contrast to an additional step of positive selection, performed in the study by Chang et al. (55), which could lead to an activation of this cell population. Whether this difference is the source of the observed discrepancy remains to be proved by additional studies. Interestingly, corroborating with our findings, a recent work, using also direct staining of peripheral blood mononuclear cells (PBMCs), reports that 2% of CD3+ T lymphocytes (2% of CD4+ and 6% of CD8+ cells) express BCMA. Additionally, 7% of circulating monocytes (CD14+) and 49% of NK cells (CD54+) also express BCMA, while TACI was undetectable from the surface of all assayed cell populations (56). The authors further report an increase in BCMA expression, on all PBMC categories in systemic lupus erythematosus (SLE) patients.

TACI is also expressed in macrophages (57, 58), being the main receptor mediating BAFF and APRIL actions, and is related to M1 polarization and the expression of a pro-inflammatory phenotype, protection from parasite infections, insulin signaling, and glucose dysregulation. Interestingly, the majority of TACI protein remains intracellular, at the perinuclear space, and is translocated to the membrane after BAFF incubation. However, the authors note an additional M1-related interleukin (IL)6 secretion by APRIL (but not BAFF) in TACI-knockout (KO) mice, attributed to a possible implication of BCMA, which, albeit absent in flow cytometry, was found at the mRNA level in mouse peritoneal macrophages (57). Interestingly, a recent work reports that circulating human plasmacytoid dendritic cells (pDCs), which represent ~0.5% of PBMCs (CD304+), transcribe selectively BCMA at levels far higher than any other circulating cell and express it on their membranes after Toll-like receptor (TLR)7 and TLR9 stimulation (59). These cells are key players in maintaining the balance between an efficient defense and preventing autoimmune responses, they have been implicated in immune tolerance and inflammation and are part of the microenvironment of tumors (60–63), as reviewed by Swiecki and Colonna (64). By expressing BCMA, these cells may therefore be, together with macrophage-expressing TACI, major players in the regulation of tumor microenvironment and might be responsible, at least in part, for an effect in tumor-induced inflammation and metastasis.

BAFF and APRIL have been reported to be trophic factors in lymphocyte malignancies and immune-related disorders (65), while they have been identified in bronchial tissue (66) and a number of immune-related and immune-independent normal and cancer tissues (spleen, liver, lung, heart, intestine, kidney, thymus, breast) (67), although the exact cellular population contributing to their expression was not clearly identified. Indeed, in tumors, BAFF and APRIL could be synthesized and secreted by B or T tumor-infiltrating lymphocytes, dendritic cells, or other components of the tumor microenvironment (68) or the tumor cells (see following chapters).

Binding of APRIL or BAFF to their respective receptors triggers diverse signaling pathways, including the activation of caspases, the translocation of NF-κB, or the activation of mitogen-activated kinases JNK or extracellular signal-regulated kinase (ERK) [see Hatzoglou et al. (69) for a specific effect in different cell lines and Mackay et al. (36) and Bossen and Schneider (33) for reviews]. However, the signaling cascades, initiated by these two ligands, seem to be tissue-specific. Indeed, in HEK293 cells, transiently or stably transfected with BCMA, we have reported that APRIL induces the canonical NF-κB pathway, together with JNK, p38, and ERK kinases (69), while, in hepatocellular carcinoma cell lines (HepG2 and Hep3B), activation of BCMA leads to the activation of a novel pathway, involving JNK2-FOXO3A-GADD45, leading to cell cycle arrest and a modest decrease of proliferation (70). In B lymphocytes, BAFF, acting through the whole spectrum of receptors, induces canonical and non-canonical NF-κB activation (71), leading to B-cell maturation (72). Similarly, TACI leads to the activation of the canonical NF-κB pathway (73) and a sustained Blimp-1 expression (53) in B cells, enhancing differentiation to long-lived antibody-secreting cells. All three receptors (BAFFR, BCMA, and TACI) can prevent apoptosis, decreasing the proapoptotic protein Bim, through a pathway implicating MAPK kinase (MEK)-ERK kinases (74). Additionally, BAFFR through the phosphoinositide 3-kinase (PI3K)-AKT/mammalian target of rapamycin (mTOR) axis activates protein synthesis, as a result of small ribosomal subunit protein S6 and the translation inhibitor 4EBP1 phosphorylation, and enhances mitochondrial function due to MLC-1 stabilization, leading to an increased cell life span (75–78). The activation of TACI and BCMA in Hodgkin lymphoma cells, through NF-κB, enhanced Bcl-2, Bcl-xL, and c-Myc and induced Bax downregulation, promoting the proliferation and survival of these cells (79).

## APRIL–BAFF and Their Receptors in Hematologic Malignancies: Therapeutic Implications

As discussed in the previous paragraph, BAFF and APRIL primarily promote cell proliferation and survival by inducing the expression of antiapoptotic molecules, regulating protein synthesis and energy metabolism. Moreover, a large number of studies revealed a significant role of BAFF–APRIL system in hematologic malignancies.

Early studies from our group identified BCMA on the membrane and the perinuclear region of lymphoid B and myeloma cells (30, 44, 69, 80). Others have also verified these results, reporting the expression of BCMA (42, 81–85), TACI (86–89), and BAFFR (42, 90) in multiple myeloma. These observations led investigators to study elements of the BAFF–APRIL system as potential therapeutics in hematologic malignancies: Anti-APRIL antibodies have been tested *in vitro* and in experimental animals in B cell lymphomas (91, 92) and multiple myeloma cells and xenografts (93–95); anti-BAFFR antibodies have been studied in multiple myeloma (96–98) with moderate results, alone or in combination with proteasome inhibitors. In contrast, anti-BAFFR antibodies was proven effective in acute (99) or chronic lymphocytic leukemia (100). Finally, targeting of TACI with either antibodies or chimeric antigen receptor (CAR) T cells was found beneficial in multiple myeloma (95, 101, 102).

The expression of BCMA preferentially in maturating cells of B- origin (85, 103), together with its reported low expression in different normal human tissues, positions the APRIL/BCMA as a prominent target for multiple myeloma treatment. Indeed, anti-APRIL antibodies or BCMA downregulation significantly decreases myeloma cell viability and colony formation (94). This element positions APRIL, autocrinally produced by these cells or paracrinally provided by stromal cells or neutrophils (104), as a primary factor in myeloma control. However, it is BCMA control which has been retained as a compelling therapeutic target in myeloma, with a limited risk of off-tissue toxicity (105). In 2013, the first report of an anti-BCMA CAR-expressing T (CAR-T) cell was published (85), promoting BCMA as a target for multiple myeloma treatment. This report was followed by an enhanced interest, propelling anti-BCMA antibodies or CAR-T cell production in the third place of therapeutics development in 2019 (106), with 16 running clinical trials, ranging from Phases I to III [reviewed in Mullard (107)], and involving CAR-T cells, monoclonal antibodies, and antibody–drug conjugates. The first reported trials with CAR-T cells (108, 109) and monoclonal antibodies (110) showed promising results. In two very recent reviews (111, 112), the authors report a good success rate of anti-BCMA CAR-T therapies. However, a high relapse rate, hematological toxicity, cytokine release syndrome, and neurological toxicity are the most prominent side effects in CAR-T treatment, while hematological toxicity and corneal events were reported in the monoclonal trial, and the duration of remission has not been resolved until now. Nevertheless, although it is early to conclude, BCMA seems to be a prominent target against multiple myeloma (113–115).

## APRIL–BAFF and Their Receptors in Solid Tumors

Since its discovery, APRIL was found to be expressed, in addition to cells of the immune system, in other tissues, including the prostate, colon, spleen, and pancreas (25). It was reported that APRIL and BAFF were also detected in bone marrow stromal cells and osteoclasts (116), while BAFF was also found in the placenta, heart, lung, fetal Iiver, thymus, and pancreas (28). BAFF was also expressed in adipocytes (117) where, in addition to its effects in adipogenesis (117), it exerts a negative modulation of the insulin receptor sensitivity (58, 118). Such actions has positioned BAFF as an adipokine, with a possible role in diabetes and obesity [reviewed in Rihacek et al. (119) and references herein].

During tumor development, inflammation in the tumor microenvironment (TME) can be a potent promoter of tumor initiation, promotion, and progression (120). During inflammation, different mediators, produced by either tumor cells or supplied by TME-infiltrating cells, account for complex interactions, influencing differentiation, activation, function, and survival/apoptosis. Targeting tumor inflammation is therefore a possible way in combatting cancer. However, all established immune-related therapies target immune cells (resident or infiltrating the tumor stroma) (121), leading to an immune checkpoint blockade (122), while the cancer cell immune-related properties and their regulation are less well-defined (123, 124). Several molecules involved in immune interactions, including the TNF superfamily members TNF, Fas, and TNF-related apoptosis-inducing ligand (TRAIL) and their receptors, have been actively investigated and targeted in a number of malignancies (121, 125). Equally, since BAFF, APRIL, and their receptors were also found in several tumor cells, their expression could represent a possible therapeutic target. Indeed, some initial efforts were done either with soluble mutant APRIL (126, 127) or with soluble BCMA molecules (128), with promising results.

APRIL transcripts were reportedly elevated in the colorectal adenocarcinoma SW480, the Burkitt's lymphoma Raji, and the melanoma G361 cell lines (25), while we have reported that about half of the most commonly used glioblastoma cell lines overexpress APRIL and BCMA (129). In addition, APRIL mRNA was found elevated in thyroid carcinoma and in lymphoma (25), as well as in colorectal tumors, as compared to non-tumoral tissue (25). Although APRIL was found to enhance the proliferation of different cancer cell lines (25), BAFF was reported to either enhance cell proliferation (28, 29) or increase apoptosis (130). Additionally, our group has reported APRIL and BAFF expression in human epithelial breast cancer cells (see below). However, although BAFF and APRIL have been advanced as possible targets in non-hematologic malignancies [reviewed in Ryan and Grewal (131) and Rihacek et al. (119)], no systematic detection of APRIL–BAFF and their receptors has been performed in different cell lines and human tumors.

**Figure 3:**
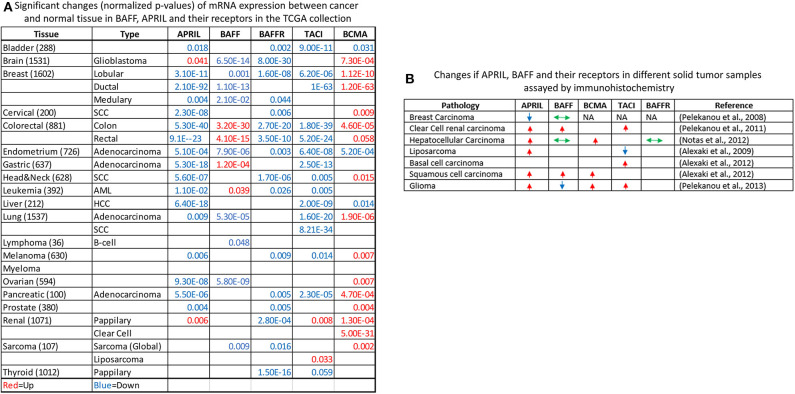
Exploration of The Cancer Genome Atlas (TCGA) collection for the expression of a proliferation-inducing ligand (APRIL), B cell-activating factor (BAFF), and their receptors **(A)**. The normalized *p*-value of the comparison of tumor vs. normal mRNA expression in all TCGA sample collection is presented, stratified by tissue of origin and disease pathology. Upregulation of mRNA expression in tumors is shown in red, while downregulation is shown in blue. Non-significant results are not shown. The Oncomine online resource (137) was used for the identification and calculation of significance. **(B)** Immunohistochemical detection of APRIL–BAFF and their receptors in human malignancies according to our work (relevant publications are shown in parentheses). Arrows denote the increased (red, up-pointing arrow), decreased (blue, down-pointing arrow), or no change (green, double-headed arrow) of proteins in immunohistochemically stained slides or tissue microarrays, with specific antibodies. NA, not detected. Please refer to the corresponding publications for further details.

An initial report by Mhawech-Fauceglia et al. (132) suggested that APRIL and BAFF were produced by the tumor stroma and more specifically by infiltrating neutrophils. However, an alternative source of these molecules might be the tumor cells themselves. In addition, the expression of BAFF–APRIL receptors by the tumor itself was not investigated in depth. Our group has undertaken the exploration of the BAFF–APRIL system in solid human malignancies (70, 117, 133–136). Our collective results are presented in Figure 3B; they show that, in the majority of pathologies we have examined, tumor cells express members of this family of ligands and receptors, whose expression is mainly enhanced in the tumor as compared to adjacent non-tumoral cells.

**Figure 4:**
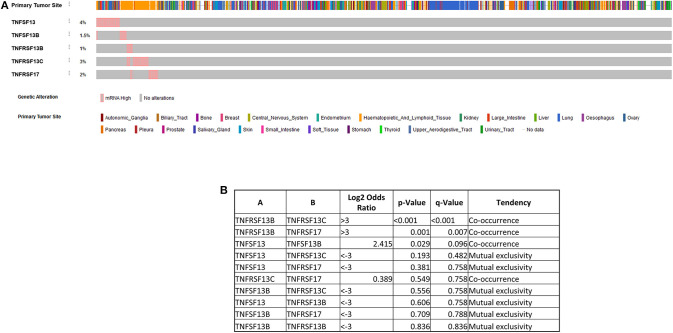
Expression of a proliferation-inducing ligand (APRIL), B cell-activating factor (BAFF), BAFF receptor (BAFFR), TNFR homolog transmembrane activator and Ca^2+^ modulator (CAML) interactor (TACI), and B cell maturation antigen (BCMA) in the Cancer Cell Line Encyclopedia (141), reporting the RNAseq data of 1,156 human cancer cell lines **(A)** using the cBioPortal web resource (139, 140). A pink bar denotes the presence of the corresponding ligand mRNA, while a gray one denotes the absence of mRNA for the corresponding ligand/receptor. Note that a co-occurrence of ligands and receptors is not always evidenced, as also shown in the Table presented in **(B)**.

In order to further explore the possible role of APRIL–BAFF and their receptors in solid tumors, we have investigated The Cancer Genome Atlas (TCGA) collection using the Oncomine resource (137) and compared tumor vs. normal mRNA expression (Figure 3A), in the whole spectrum of the samples' collection. Interestingly, specimens of all locations express members of the BAFF–APRIL system of ligands and receptors. However, with the exception of BCMA, which is usually upregulated in tumor samples as compared to their non-tumoral counterparts, all other members are usually downregulated in tumors as compared to normal specimens. However, this result should be interpreted with caution, as the TCGA collection comprises stromal immune cell infiltrates at different proportions, ranging from 5 to >60% (138). Moreover, using the cBioPortal web resource (139, 140), we analyzed the expression of APRIL, BAFF, BAFFR, TACI, and BCMA in the Cancer Cell Line Encyclopedia (141), reporting the RNAseq data of 1,156 human cancer cell lines. We found that the expression of these proteins is restricted in very few cell lines (<5%), mainly in cell lines deriving from hemopoietic and lymphoid tissues (Figure 4). Curiously, when we examined the co-occurrence of these molecules on the same cell line, we did not find a hard evidence of a co-expression of ligands and receptors, suggesting perhaps that the mode of action of this system might not occur through an autocrine manner. However, APRIL and BCMA expression was further found in cells of other origins, including the breast. Finally, it is to note that, until now, no trial implicating the APRIL–BAFF system has been initiated for solid tumors.

## APRIL–BAFF System in the Breast

As in every solid tumor pathology, the breast tumor microenvironment plays a prominent role in tumor initiation, promotion, and progression (120). Local inflammation is, indeed, a common element in the initiation and progression of breast cancer, and a number of innate (macrophages) or infiltrating lymphocytes have been reported (142), which are modified by chemotherapy or personalized immune therapy (143). In an analysis of the TCGA data, with an immune score for different infiltrating cell types (144), it was found that macrophages and neutrophil infiltrates [which express TACI and APRIL, respectively (57, 58, 104)] are related to poor prognosis in breast cancer. Interestingly, stromal macrophages, a population implicated in breast cancer evolution (145–147) are significantly decreased by therapy (148). Macrophages express TACI (57, 58), which is related to M1 pro-inflammatory polarization. In addition, as shown in Figure 2, circulating monocytes, which could migrate in sites of inflammation, express, in addition to TACI, BCMA, together with plasmatocytoid dendritic cells, expressing BCMA (59). It might therefore be interesting to investigate the possible phenotypic changes of monocyte to macrophage switch and its possible implication in the pro-inflammatory role of macrophages' APRIL–BAFF system, in the breast stroma.

In 2008, we presented the first evidence for the expression of APRIL and BAFF in specimens of breast cancer patients and reported the expression of these molecules in human epithelial breast cancer cells (133), repositioning the established notion that the major source of APRIL was either stroma cells (68) or infiltrating neutrophils (132). Our data showed that BAFF was ubiquitously expressed by non-cancerous and cancerous breast epithelia, while its expression was not modified by tumor evolution. In addition, BAFF was expressed also by stromal cells and breast adipocytes. It is to note that further research of our group confirmed this finding, by identifying APRIL and BAFF and their receptors in normal and neoplastic adipose tissue (117). However, APRIL expression is decreased during adipocyte differentiation, while the ligand reappears during tumorigenesis. In the breast, APRIL was preferentially expressed by the non-cancerous breast epithelial tissue, while its expression was decreased in breast tumoral cells. APRIL expression was antiparallel with the tumor grade, while it was preferentially expressed by node-positive as compared to node-negative tumors (133). Neither BAFF nor APRIL was related to patients' outcome [disease-free survival (DFS) or overall survival (OS)].

The role of APRIL in breast cancer was further analyzed by Garcia-Castro et al. (149). The authors confirmed the expression of APRIL, as well as its receptors BCMA and TACI, in breast cancer cell lines MCF7, T47D, MDA-MB-231, and MDA-MB-468. Interestingly, BCMA and TACI, although present in all four cell lines were highly expressed in the mesenchymal-like MDA-MB-231 and MDA-MB-468 cells, suggesting a possible role in tumor aggressiveness. This was further supported by the preferential expression of APRIL and its receptors in a small series of triple-negative human tumors as compared to luminal-type carcinomas (149). Finally, the authors reported a direct effect of APRIL on cell proliferation [reported also by other groups (150, 151)] and the induction of tumor xenografts in an APRIL-rich environment. Interestingly, in a recent work (151), we have reported that APRIL is upregulated by membrane-acting androgen, and that induces breast cancer cell migration and epithelial-to-mesenchymal transition, together with mammosphere formation and induction of stemness. This action was mediated by BCMA, positioning this receptor as a possible therapeutic candidate in breast cancer (see the following paragraph). The effects of APRIL in the breast seem to be exclusively mediated through the JNK pathway (149, 151).

In a recent work, Abo-Elfadl et al. (152) explored the silencing of TACI in breast cancer cell lines. The authors reported a significant inhibition of cell viability and induction of apoptosis, mediated through an inhibition of the Bcl-2 protein. They suggested that, similarly to BCMA, TACI may also have a potential role in breast cancer treatment.

**Figure 5:**
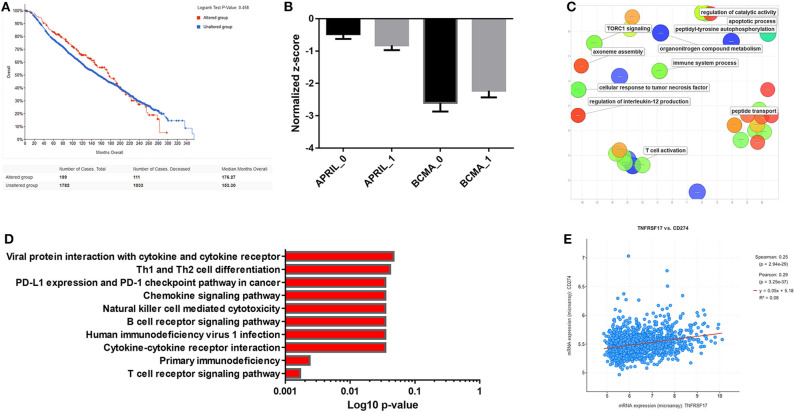
**(A)** Survival curves of a proliferation-inducing ligand (APRIL)-, B cell maturation antigen (BCMA)-, and TNFR homolog transmembrane activator and Ca^2+^ modulator (CAML) interactor (TACI)-positive breast tumor patients from the METABRIC study (153, 154) analyzed by the cBioportal for cancer genomics (139, 140). **(B)** Expression of APRIL and BCMA before (0) and after treatment (1) of breast tumors in the dataset GSE114403 (148). Mean ± SE is presented. **(C)** BCMA-co-regulated genes in the METABRIC study were extracted, and Gene Ontology (GO) terms (MF) were analyzed with g:Profiler (155). Significant terms were plotted with REVIGO (156). **(D)** Kyoto encyclopedia of genes and genomes (KEGG) pathway analysis of BCMA-co-regulated genes from the METABRIC study, analyzed with g:Profiler. **(E)** Correlation of BCMA with programmed cell death ligand (PDL)-1 (CD274) expression in the METABRIC study.

In an attempt to further investigate the role of the APRIL–BAFF system in breast cancer, we have concentrated on the role of APRIL and its receptors BCMA and TACI, as APRIL is the ligand found to be differentially expressed in breast cancer (133), and BCMA and TACI were found also in breast cancer cells (149, 151, 152). We have investigated the METABRIC dataset (153, 154). Using the cBioportal for cancer genomics (139, 140), we confirm that the modification of either APRIL or its receptors do not have an effect on survival (Figure 5A). This is further corroborated by the analysis of dataset GSE114403, investigating the effect of treatment on breast cancer inflammatory genes (148) and found that treatment induces a slight decrease in APRIL and a slight increase in BCMA (Figure 5B). We have further extracted the co-regulated genes with APRIL, TACI, and BCMA, in the METABRIC study, in cBioportal, and retained the 1,758 significantly co-modified gene signatures (*p*-value cutoff 0.001). Significant Gene Ontology (GO) terms were extracted with g:Profiler (155), and significant terms were plotted with REVIGO (156). Biological processes terms (Figure 5C) show that APRIL and its receptor changes modified terms related to T cells, cellular response to cytokines and TNFR, and processes related to apoptosis. Interestingly, in Kyoto Encyclopedia of Genes and Genomes (KEGG) pathway analysis (Figure 5D), significant interactions were found also with the programmed cell death ligand (PDL)-1 and programmed cell death (PD)-1 checkpoint in cancer. This was further verified by the significant positive correlation between BCMA and PDL-1 (CD278; Figure 5E).

## Possible Novel Therapeutic Modalities Involving BCMA and TACI in Breast Cancer

As discussed above, only scarce data exist about the role of APRIL and its receptors in breast cancer. However, breast cancer is one of the few tumors characterized by a differential role of BAFF and APRIL, with BAFF being constantly present in both normal and tumor tissue, and APRIL being expressed in a completely different manner in normal and tumorous breast tissue. There is a concordance regarding the role of APRIL in this malignancy, suggesting that this cytokine can actually promote the survival, proliferation, and migration of breast cancer cells (149–152) in accordance with the pro-proliferative action of APRIL in epithelial tumors and hematological malignancies (79). It is to note that the only tissue in which APRIL, through BCMA, was reported to inhibit cell proliferation is the liver (70). Concerning involved receptors, BCMA seems the most prominent candidate. Indeed, BCMA has been reported to mediate APRIL effects either alone (151) or in combination with TACI (149). However, the role of TACI in breast cancer is not very clear so far. The analysis of the TCGA data shows a decrease of TACI in tumors as compared to non-tumoral tissue in contrast to BCMA which is upregulated in both lobular and ductal carcinomas (Figure 3A), although two groups suggest that TACI might be an interesting target in breast cancer (149, 152).

Recently, in addition to anti-BCMA biological therapies in hematologic malignancies (discussed above), antibodies against TACI (157) or BAFF (158) have been tested in immunological diseases, with variable results. Therefore, BCMA-targeting treatments might be a more advanced target in breast cancer, especially as BCMA seems to correlate with an established checkpoint molecule, PDL-1 (see the previous paragraph and Figure 5E). Indeed, as shown in Figures 3A,B, BCMA is upregulated in a large number of tumors, outside of lymphomas and leukemias, including breast cancer. However, there are significant issues that need to be clarified before the introduction of such a therapy in solid tumors:

**First**, the role of APRIL and its receptors is far from being elucidated. For example, APRIL seems to be upregulated by androgen (151), TLR3-induced inflammation (149), or modulation of its receptors (152), inducing either enhancement or reduction of cell proliferation, migration, and stemness, mediated by BCMA (70, 149, 151).

**Second**, the presence of both APRIL and its receptors in an array of normal tissues (Figure 3) suggests a possible important physiological effect of this system, which should be further investigated before the application of any biological therapy. For example, unpublished data from our group indicate that BCMA is differentially expressed in normal testis in relation to sperm production and maturation, while we have reported a direct inhibitory effect of APRIL in adipogenesis (117) and a pro-inflammatory action in the skin (135).

These and other, not yet identified, effects of APRIL/BCMA, together with the reported toxicity, cytokine release syndrome and neurological toxicity, after the application of anti-BCMA therapies in hematologic malignancies, discussed above, suggest that extreme caution should be exercised before initiating relevant clinical studies in breast cancer.

## Data Availability Statement

All datasets for this study are included in the article/supplementary material.

## Author Contributions

All authors listed have made a substantial, direct and intellectual contribution to the work, and approved it for publication.

## Conflict of Interest

The authors declare that the research was conducted in the absence of any commercial or financial relationships that could be construed as a potential conflict of interest.
